# The Effects of Dietary Glycerol Fatty Acid Esters on the Production Performance, Serum Biochemistry, and Rumen Microbial Community of Crossbred Simmental Bulls

**DOI:** 10.3390/ani15152194

**Published:** 2025-07-25

**Authors:** Lei Yang, Shijun Tian, Yongchang Luo, Zhanhong Qiao, Chao Chen, Xiaokang Lv, Jinling Hua

**Affiliations:** 1 College of Animal Science, Anhui Science and Technology University, Chuzhou 239000, China; yanglei2025001@163.com (L.Y.);; 2 Anhui Province Key Laboratory of Animal Nutritional Regulation and Health, Chuzhou 233100, China

**Keywords:** glycerol fatty acid esters, production performance, serum biochemical parameters, rumen microbiota

## Abstract

Glycerol fatty acid esters (GFAEs) are recognized for their potential to improve lipid metabolism, energy utilization, and gut health due to their excellent emulsifying and antimicrobial properties. The objective of this research was to investigate the effects of dietary GFAE supplementation on production performance, serum biochemical profiles, and rumen fermentation in beef cattle. Thirty crossbred Simmental bulls, averaging 507.42 ± 9.59 kg in body weight, were assigned to three distinct cohorts, with 10 animals in each cohort. The CON cohort was fed a basal diet devoid of GFAE, whereas the treatment cohorts (GFAE1 and GFAE2) received GFAE supplements at concentrations of 0.1% and 0.2% of the dietary dry matter, respectively. Compared with the control group, supplementation with 0.1% GFAE significantly increased the ADG of beef cattle by 12.14% (*p* < 0.05); compared with the GFAE2 group, ADG was 7.86% higher (*p* > 0.05). The digestibility of NDF and ADF was significantly enhanced in the GFAE1 group relative to the control group (*p* < 0.05). Dietary GFAE supplementation significantly elevated rumen acetate, propionate, and total volatile fatty acid concentrations in both the GFAE1 and GFAE2 groups compared to the control group (*p* < 0.05). In contrast to the control group, there was a notable rise in serum levels of T-AOC, UREA, and TG in both GFAE1 and GFAE2 groups (*p* < 0.05). Conversely, the concentration of HDL-C was significantly decreased in the GFAE2 group. Additionally, at the phylum level, the abundance of Fibrobacterota was significantly higher in the GFAE1 group than in the control group (*p* < 0.01). At the genus level, the proportions of *Bacteroides* and *Fibrobacter* were significantly higher in the GFAE1 group compared to the control group (*p* < 0.05). In conclusion, this study demonstrates that the addition of 0.1% GFAE to beef cattle diets significantly enhances the digestibility of ADF and NDF nutrients, increases serum total antioxidant capacity, urea, and triglycerides, optimizes rumen fermentation parameters and microbial community structure, and ultimately improves production performance. As a result of the findings from this research, it is suggested that 0.1% GFAE be incorporated into the diet for beef cattle.

## Simple Summary

Glycerol fatty acid esters (GFAEs) are increasingly recognized as promising dietary additives for ruminants, owing to their potential to boost growth, enhance nutrient digestion, and modulate rumen fermentation. The present research sought to examine the influence of providing 0.1% and 0.2% GFAE through feed on the output, blood biochemical characteristics, and the composition of the rumen’s microbial population in Simmental crossbred steers. The results indicated that dietary inclusion of 0.1% GFAE significantly enhanced serum antioxidant capacity, fiber digestibility, and rumen fermentation parameters, alongside an elevated average daily gain. Furthermore, a significant elevation in the proportional representation of both the Fibrobacterota phylum and the *Bacteroides* and *Fibrobacter* genera was observed. These results indicate that the addition of an appropriate amount of GFAE to the diet can enhance the production performance of beef cattle without having a negative impact on their health.

## 1. Introduction

Driven by the increasing global population and the continuous rise in demand for meat products, the beef cattle industry plays a vital role in ensuring meat supply [1,2]. Numerous studies have demonstrated that the use of feed additives during the finishing process can enhance the production performance and efficiency of livestock and poultry [3,4]. In recent years, owing to advancements in animal nutrition research, an increasing number of novel feed additives have been developed and applied in beef cattle farming. These additives aim to improve the nutritional value and utilization rate of feed, thereby enhancing the production performance of beef cattle. Moreover, the rational application of these novel feed additives is crucial to ensure feed safety and comply with stringent animal hygiene standards, thereby contributing to the transformation and upgrading of the livestock industry.

Glycerol fatty acid esters (GFAEs) are compounds generated through the esterification reaction between glycerol and fatty acids, possessing properties such as emulsification, antimicrobial activity, and energy supply [5]. With a wide variety of types, GFAEs can be classified into short-chain, medium-chain, and long-chain GFAEs based on the type and carbon chain length of the fatty acids [6]. In the field of feed additives, GFAEs can regulate animal nutrition metabolism and improve gut health, thereby enhancing feed utilization [7,8]. In recent years, as a novel feed additive, GFAE has been widely applied in livestock production [7]. Due to their unique molecular structure, GFAEs rapidly provide energy, exhibit broad-spectrum antimicrobial activity, and modulate gut microbiota composition [9]. Previous studies have demonstrated that GFAE significantly enhances both the growth performance and gut health of livestock and poultry [10,11]. Especially in rearing methods that do not use antibiotics, the efficacy of GFAE as a substitute for antibiotics has been validated in trials involving broilers and piglets, showing notable results in promoting gut health and bolstering immune function [12,13]. The antimicrobial properties of GFAEs are primarily attributed to their ability to disrupt the cell membranes of microorganisms [14]. Medium-chain GFAEs, in particular, have been shown to effectively inhibit the growth of Gram-negative and Gram-positive bacteria by increasing membrane permeability and causing leakage of cellular contents [15]. Research by Masmeijer et al. [16] demonstrated that adding GFAEs to the diets of calves significantly improved immune regulation, which was reflected in the reduced production of reactive oxygen species and elevated secretion of proinflammatory cytokines. Similarly, Vinolo et al. [17] highlighted that GFAEs play a significant role in modulating inflammatory responses. Based on these findings, we hypothesize that supplementing beef cattle diets with GFAEs will enhance feed efficiency, improve rumen health, and modulate immune responses. Further research is warranted to elucidate the mechanisms of action, determine optimal dosages, and assess the long-term effects of GFAE supplementation on beef cattle production, despite the preliminary evidence of its potential benefits.

The purpose of this study was to investigate the effects of adding GFAE based on various factors in beef cattle, such as nutrient digestibility, production performance, apparent biochemical parameters in the serum, characteristics of rumen fermentation, and the composition of rumen microbiota. The results aim to provide a scientific basis for the use of GFAE in beef cattle production.

## 2. Materials and Methods

### 2.1. Research Location and Materials

The feeding trial took place from October to December 2024 at Zhangpu Farm of Henan Hengdu Xia Nan Cattle Development Co., Ltd., Henan Province, China. GFAEs were obtained from Guangzhou Biorule Co., Ltd., Guangzhou, China, and were in the form of light gray–brown powder or granules. The main components are small-molecule α-monolaurin glycerides and metabolites of Lactobacillus. The premix containing concentrated feed was purchased from Cargill Animal Nutrition (Zhengzhou) Co., Ltd., Zhengzhou, China.

### 2.2. Experimental Design

Thirty crossbred Simmental bulls with similar age (15–16 months) and weight (507.42 ± 9.59 kg) were randomly assigned to three distinct cohorts, with 10 animals in each cohort. The bulls were group-housed in pens, with each cohort occupying a separate pen. The CON cohort was fed a basal diet devoid of GFAE, whereas the treatment cohorts (GFAE1 and GFAE2) received GFAE supplements at concentrations of 0.1% and 0.2% of the dietary dry matter, respectively. The foundational diet was formulated based on the nutritional needs outlined in the Feeding Standard for Beef Cattle (NY/T 815 [18]). Feeding of the cattle occurred two times per day, specifically at 08:00 and 16:00, and they had continuous access to water for the entire duration of the study. The study extended over a duration of 66 days, which included a 6-day adaptation period. Cleaning and disinfecting the barn were conducted in accordance with the farm’s standard management protocols. Table 1 presents the specific components and nutritional values of the basal diet.

### 2.3. Production Performance

Before the official experimental phase began, the cattle were weighed following an overnight fast to assess their IBW. The FW was measured on day 60. ADGs were calculated based on the initial and final body weights. Throughout the entire experimental period, the weights of the feed provided and the leftover feed for each group were documented daily to calculate the DMI. The F/G was calculated based on DMI/ADG.

### 2.4. Apparent Nutrient Digestibility

Fecal sampling: The sampling time points were 08:00 and 14:00 daily. From Day 57 to Day 59 of the experiment, nine cattle were selected from each of the experimental groups to collect fecal samples from the rectum. A total of 600 g of feces was collected from each animal per day, mixed thoroughly, and divided into two portions. One portion was stored without acid, while the other portion was treated with 10% sulfuric acid solution (20 mL acid per 100 g feces) and kept at −20 °C for later analysis. Crude ash and DM were determined using the method of Zhang et al. [19]. The measurements for ADF and NDF were carried out using the procedure outlined by Van Soest et al. [20]. The analysis of CP was conducted using a Kjeldahl 8400 analyzer unit (Foss, Beijing, China) according to the method described by Zhang et al. [21], while the determination of EE followed the standard GB/T 6433-2006 [22]. For measuring acid-insoluble ash (AIA), the standard GB/T 23742-2009 [23] was utilized as a reference in determining AIA in feed. Apparent digestibility of nutrients = 100 − [(a/b) × (c/d)] × 100, where a represents the content of AIA in the diet, b represents the content of AIA in the feces, c represents the content of the nutrient in the feces, and d represents the content of the nutrient in the diet.

### 2.5. Serum Biochemical Parameters

On the morning of the 60th trial day, blood samples (10 mL) were collected from the jugular vein at 06:00, prior to the morning feeding scheduled at 08:00. The samples were then immediately centrifuged at 3000 rpm for 15 min to isolate the serum. Subsequently, the serum samples were immersed in liquid nitrogen and later stored at −80 °C. The subsequent determination of serum-related parameters was conducted by Nanjing Aoxing Biotechnology Co., Ltd., Nanjing, China.

### 2.6. Rumen Fluid Sample Collection and Analysis

On day 60, rumen fluid was then collected through the oral cavity after a fasting period. The collected fluid was filtered using four layers of gauze and separated into three aliquots. One aliquot was used immediately to determine the pH using a portable pH meter (model S220-K, Mettler Toledo, Shanghai, China). The remaining two portions were immersed in liquid nitrogen for quick freezing and subsequently kept at −80 °C for future analysis. The level of NH3-N in the rumen fluid was determined using a spectrophotometer (model TU-1901, Beijing Puxi General Instrument Co., Ltd., Beijing, China) based on a colorimetric method. Volatile fatty acids (VFAs) were analyzed by gas chromatography (A91Plus, Pano Instrument Co., Ltd., Changzhou, China) in accordance with the procedure outlined by Zhao et al. [24].

### 2.7. Rumen Microbiota Analysis

In accordance with the description in Supplementary Method S1, 12 frozen rumen fluid samples, with 4 samples per group, were sent to Majorbio Bio-Pharm Technology Co., Ltd., Shanghai, China, for metagenomic sequencing analysis.

### 2.8. Data Statistics and Analysis

All statistical analyses were performed using SPSS 25.0 software. Prior to analysis of growth performance, nutrient digestibility, serum biochemical parameters, and rumen fermentation parameters, data were assessed for normality and homogeneity of variance using the Shapiro–Wilk test and Levene’s test, respectively. Data meeting the assumptions of normality and homogeneity of variance were subjected to one-way ANOVA to determine the effects of GFAE supplementation. When significant differences were detected (*p* < 0.05), Duncan’s multiple range test was used for post hoc pairwise comparisons. Differences in bacterial compositions were analyzed using the Kruskal–Wallis test, followed by Dunn’s post hoc test for pairwise comparisons with Benjamini–Hochberg correction for multiple comparisons. A *p*-value of less than 0.05 (*p* < 0.05) was considered statistically significant. In the data, values marked with different superscript letters indicate significant differences (*p* < 0.05).

## 3. Results

### 3.1. Production Performance of Beef Cattle

As shown in Table 2, compared with the control group, supplementation with 0.1% GFAEs significantly increased the ADG of beef cattle by 12.14% (*p* < 0.05); compared with the GFAE2 group, ADG was 7.86% higher (*p* > 0.05). However, IBW, FBW, DMI, and F/G did not differ significantly among the groups (*p* > 0.05).

### 3.2. Apparent Digestibility of Nutrients in Beef Cattle

Incorporating GFAE into the feed improved the digestibility of NDF and ADF in beef cattle (*p* < 0.05; Table 3). Specifically, the apparent digestibility of NDF was significantly higher in the GFAE1 group compared to the CON group (*p* < 0.05). In addition, the apparent digestibility of ADF in the GFAE1 group exceeded that of both the control and GFAE2 groups (*p* < 0.05). No significant variations were observed in the digestibility of DM, CP, and EE across the groups (*p* > 0.05).

### 3.3. Serum Biochemical Parameters in Beef Cattle

The Total Antioxidant Capacity (T-AOC) in the GFAE1 and GFAE2 groups was substantially greater than that in the CON group (*p* < 0.05). In the GFAE1 group, serum UREA and TG levels were markedly elevated compared with the CON and GFAE2 groups (*p* < 0.05). The serum HDL-C level in the GFAE2 group was notably diminished relative to the CON and GFAE1 groups (*p* < 0.05). No notable variations were detected in the serum levels of MDA, GPX, SOD, H_2_O_2_, TP, ALB, TC, LDL-C, and NEFA, across all experimental groups (*p* > 0.05; Table 4).

### 3.4. Rumen Fermentation Parameters in Beef Cattle

Supplementation with GFAE significantly increased the concentrations of acetate, propionate, and total volatile fatty acids in the rumen of beef cattle (*p* < 0.05; Table 5). Specifically, both the GFAE1 and GFAE2 groups showed markedly elevated levels of acetate, propionate, and total volatile fatty acids relative to the CON group (*p* < 0.05). However, GFAE supplementation did not significantly impact rumen pH, butyrate concentration, acetate-to-propionate ratio, or NH_3_-N levels (*p* > 0.05).

### 3.5. Overview of Rumen Microbiota in Beef Cattle

**Figure 1:**
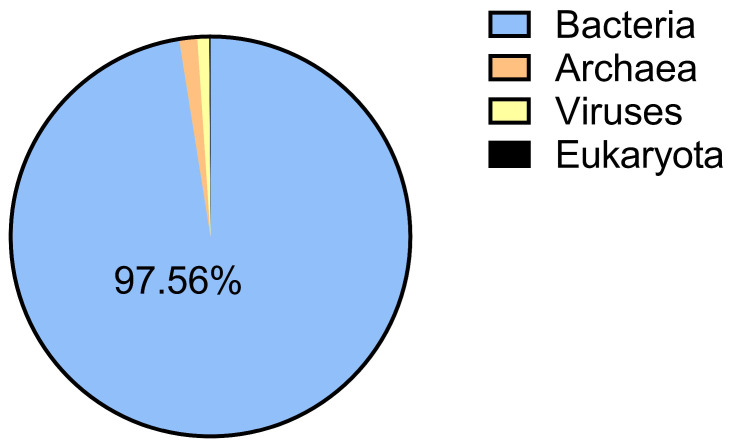
The effect of GFAE on the relative composition of rumen microbiota in finishing cattle.

A total of 519,237,914 raw sequences were obtained from the 12 metagenomic samples, corresponding to approximately 78 Gpb of bases. Species annotation was performed by aligning the non-redundant gene set to the NR database, identifying four major categories of organisms: bacteria, archaea, viruses, and fungi. Among these, bacteria accounted for the highest abundance at 97.56%, with the remainder belonging to archaea, fungi, and viruses (Figure 1).

### 3.6. Impacts of GFAE on Beef Cattle Rumen Microbiota

**Figure 2:**
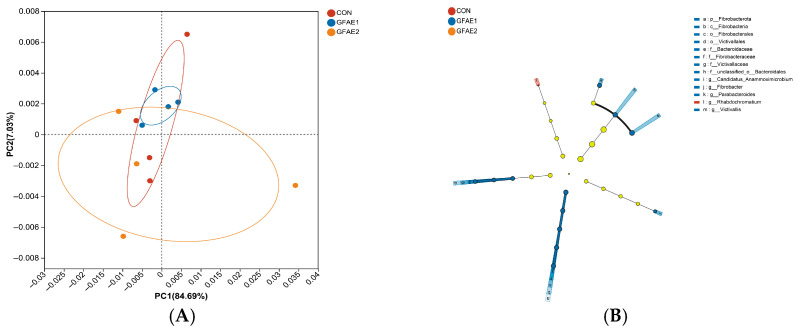
Effects of GFAE on rumen microbiota of beef cattle. (**A**) Principal coordinate analysis (PCoA) of rumen microbial communities. (**B**) Linear discriminant analysis (LDA) effect size (LEfSe) of the ruminal microbiota (Kruskal–Wallis test *p* < 0.05; Wilcoxon test among three groups *p* < 0.05; LDA score threshold > 2.0). Taxonomic rank labels are provided before bacterial names; p_, c_, o_, f_, and g_ indicate phylum, class, order, family, and genus, respectively. The greater the LDA score of the biomarker taxon mean value, the greater the influence of species abundance on the difference in the microbial community in the different treatments. CON: 0%GFAE + TMR; GFAE1: 0.1%GFAE + TMR; GFAE2: 0.2%GFAE + TMR.

Analysis of beta-diversity was performed by utilizing the NR species annotation results, and the PCoA of the samples is illustrated in Figure 2A. The PCoA indicated that samples from the CON, GFAE1, and GFAE2 groups were positioned on opposite ends of the PC1 axis, highlighting notable disparities in microbial community composition among these three treatment groups. To gain a deeper understanding of the differences in bacterial communities and to pinpoint specific biomarkers, LEfSe was employed. This evaluation recognized 13 distinct biomarkers in the microbial community (Figure 2B). Within the CON group, the genus *Rhabdochromatium* was the only one that showed significant enrichment. In contrast, the GFAE1 group exhibited significant enrichment of 12 taxonomic units, including the phylum *Fibrobacterota*, class *Fibrobacteria*, orders *Fibrobacterales* and *Victivallales*, families *Bacteroidaceae*, *Fibrobacteraceae*, and *Victivallaceae*, unclassified members of the order *Bacteroidales*, and the genera *Candidatus Anammoximicrobium*, *Fibrobacter*, *Parabacteroides*, and *Victivallis*.

**Figure 3:**
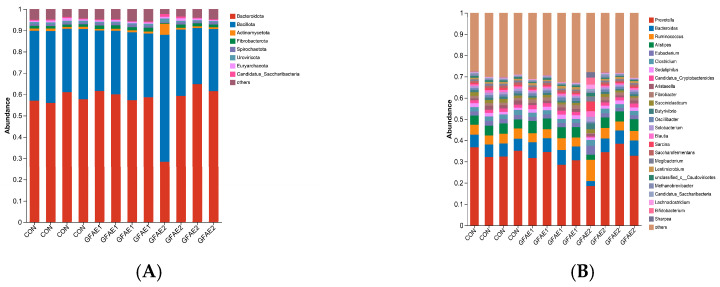
The impact of GFAE on the relative abundance of bacteria in the rumen of beef cattle. (**A**) Phylum level. (**B**) Genus level. CON: 0%GFAE + TMR; GFAE1: 0.1%GFAE + TMR; GFAE2: 0.2%GFAE + TMR. The different colors of the bars in (**A**,**B**) represent different species, and the length of the bars represents the proportion of the species.

In the analysis of microbial composition at the phylum level, Bacteroidota, Bacillota, Fibrobacterota, and Actinobacteriota exhibited relatively high abundance in all treatment groups (Figure 3A). Among them, Bacteroidota and Bacillota were dominant in all treatment groups. In contrast to the CON group, the proportion of Fibrobacterota in the GFAE1 group was notably higher (*p* < 0.01; Supplementary Table S1). Additionally, the relative abundance of Bacteroidota in the GFAE1 group showed an increase of 2% and 11% when compared to the CON and GFAE2 groups, respectively. A total of 25 genera had relative abundances exceeding 1%, including *Prevotella*, *Bacteroides*, *Ruminococcus*, *Alistipes*, *Eubacterium*, *Clostridium*, *Sodaliphilus*, *Candidatus-Cryptobacteroides*, *Aristaeella*, and *Fibrobacter*, among others (Figure 3B). As shown in Supplementary Table S2, significant variations were detected in the proportions of *Bacteroides* and *Fibrobacter* across various treatment groups (*p* < 0.05).

### 3.7. Correlation Between Rumen Fermentation Parameters and Microbial Community Composition

**Figure 4:**
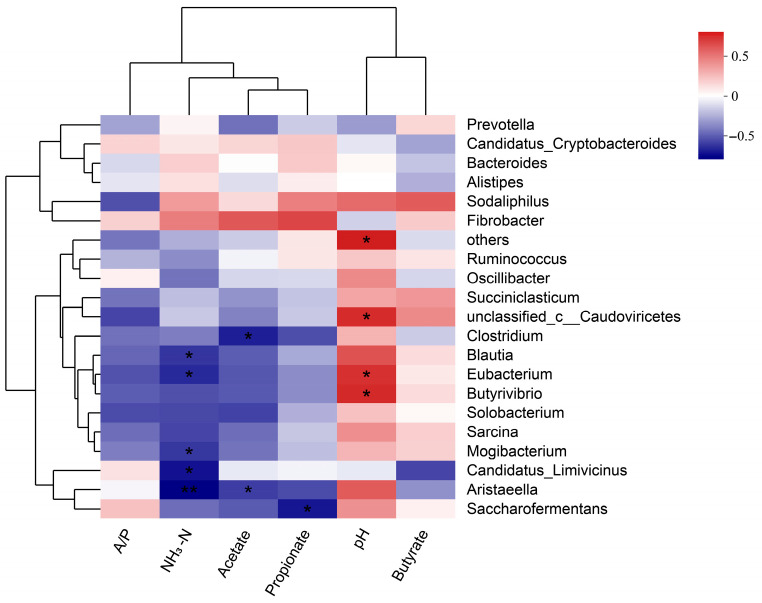
Correlation between rumen microbial communities (at the genus level) and rumen fermentation characteristics. Rumen fermentation parameters: A/P (acetate/propionate ratio); NH_3_-N (ammonia nitrogen); Acetate (acetic acid); Propionate (propionic acid); Butyrate (butyric acid). Top 20 Abundant Genera: Vertical axis indicates the relative abundance of the top 20 most abundant genera. In the heatmap, positive correlations (indicated by red block) and negative correlations (indicated by blue block) are illustrated through Spearman correlation analysis. (* indicates *p* < 0.05; ** indicates *p* < 0.01).

Spearman correlation analysis demonstrated significant associations between the abundance of key rumen bacterial genera and rumen fermentation parameters (Figure 4). Specifically, an extremely significant negative correlation was found between ruminal NH_3_-N and *Aristaeella* (*p* < 0.01), while *Mogibacterium*, *Eubacterium*, and *Blautia* also exhibited significant negative correlations with ruminal NH_3_-N (*p* < 0.05). Moreover, a marked inverse relationship was identified between rumen acetate and both *Clostridium* and *Aristaeella* (*p* < 0.05). Rumen propionate exhibited a significant inverse association with *Saccharofermentans* (*p* < 0.05). Furthermore, *Eubacterium* and *Butyrivibrio* demonstrated a significant positive relationship with rumen pH (*p* < 0.05).

## 4. Discussion

The results of this research indicated that the inclusion of different levels of GFAE in the beef cattle diet had no significant effect on DMI, which aligns with previous research. For instance, Luan et al. [25] suggested that GFAE may promote feed intake by stimulating the secretion of acylated ghrelin, implying that GFAE does not exert a negative effect on feed intake. Similarly, Righi et al. [26] reported that the addition of monoglycerides containing short-chain and medium-chain fatty acids to calf milk replacers did not significantly impact feed conversion efficiency. Additionally, Lan and Kim [27] found that adding a combination of organic acids and medium-chain fatty acids to lactating sow diets did not significantly affect weight loss, average daily feed intake, backfat thickness, dry matter digestibility, nitrogen, or energy utilization. However, although there was no significant effect on DMI, this study found significant differences in the ADG of beef cattle with different levels of GFAE supplementation. Specifically, compared to the control group, the GFAE1 group had the highest ADG, which was significantly higher than that of the control group. In contrast, the ADG of the GFAE2 group was comparable to that of the control group, with no significant difference. This result suggests that higher doses of GFAEs may not further enhance growth performance and could potentially have negative effects. This is similar to the conclusion proposed by Ee et al. [28], where excessive MCFA can lead to loss of appetite in broilers, thereby inhibiting production performance. Previous studies have indicated that the appropriate addition of GFAE may have the potential to improve animal growth performance. For example, the inclusion of MCFA mixtures in finishing pig diets has been shown to significantly increase ADG and feed efficiency, with a tendency to improve DMI [29]. Additionally, supplementation of glyceryl monolaurate in the diet of large yellow croaker has been reported to significantly enhance body weight [30]. Collectively, these findings support the potential application value of glyceride derivatives in improving animal growth performance. Building upon these findings, this study will further explore the effects of GFAE on how well diets are digested in beef cattle.

Supplementing the diet with GFAE notably enhanced how well NDF and ADF were digested in cattle during the finishing phase. Similar effects were observed by Jiang et al. [31], who reported a significant enhancement of NDF and ADF apparent digestibility in yaks with the inclusion of a 0.3% mixture of fatty acid esters in the diet. Under in vitro ruminal fermentation conditions, Luan et al. [32] found that when high-concentration feed was used as the fermentation substrate, the addition of medium-chain fatty acids significantly increased the apparent digestibility of DM and NDF. Similarly, De Souza et al. [33] showed that the inclusion of triglycerides rich in palmitic acid in the diet can enhance the digestion efficiency of DM and NDF in lactating dairy cows. GFAE may enhance fiber digestibility by modulating the rumen microbial community and promoting the proliferation of fiber-degrading bacteria. This effect is attributed to the incorporation of specific fatty acids from the diet into the bacterial cell membranes, thereby sparing energy that would otherwise be used for fatty acid synthesis and allowing for increased bacterial growth and proliferation within the rumen [34]. Consistent with this hypothesis, prior research has demonstrated that short-chain GFAEs can boost the abundance of fiber-degrading microbial communities in the rumen, thereby enhancing fiber digestibility [34]. A study in weaned piglets also found that dietary supplementation with α-glycerol monolaurate and tributyrin significantly altered the composition of the gut microbiota, thereby affecting fiber degradation and nutrient absorption [35]. Similarly, Jiang et al. [31] also observed that fatty acid ester mixtures significantly increased the relative abundance of fiber-degrading bacteria, leading to improved apparent digestibility of NDF and ADF. These results collectively indicate that GFAE might improve the digestibility of fiber by increasing the proportion of bacteria that break down fiber in the rumen.

Serum biochemical parameters can reflect the physiological condition of animals, and the levels of related hormones and immune-related markers are usually the basis for assessing the health status of experimental animals [36,37]. In the current study, dietary GFAE supplementation in beef cattle resulted in an enhancement of antioxidant capacity and elevated serum urea and triglyceride concentrations. T-AOC is a comprehensive parameter reflecting the cumulative concentration of antioxidants in serum and indicative of the body’s overall capacity to mitigate oxidative stress [38]. The elevated T-AOC observed in the GFAE group in this study is consistent with the findings of Śpitalniak-Bajerska et al. [39], who reported that supplementation with n-3 fatty acids can enhance the antioxidant capacity of calves by modulating the composition of cell membrane phospholipids and activating the Nrf2 signaling pathway. Meanwhile, this study observed that serum urea and triglyceride levels in the GFAE1 group were significantly higher than those in the control group and the GFAE2 group. Serum urea is the end product of protein catabolism, primarily synthesized in the liver and excreted by the kidneys [40]. The urea cycle converts ammonia into urea in the liver, which is an energy-consuming process [41]. Considering the observed increases in serum triglycerides and urea levels in the GFAE supplementation group, we speculate that GFAE may serve as a readily available energy source, promoting the urea cycle and thereby influencing serum urea and triglyceride levels in beef cattle. However, it is noteworthy that dietary GFAE supplementation may also reduce the serum high-density lipoprotein cholesterol (HDL-C) concentration in beef cattle, which is consistent with previous studies indicating that replacing saturated fatty acids with polyunsaturated fatty acids can lower serum TC, LDL-C, and HDL-C levels [42]. In this study, the group supplemented with 0.2% GFAE showed a significant decrease in serum HDL-C concentration. This phenomenon bears similarity to the findings of Shokrollahi et al. [43] in broilers, who discovered that the addition of medium- and short-chain glycerol fatty acid esters could inhibit the production of rate-limiting enzymes in cholesterol synthesis, thereby reducing blood cholesterol levels. Low serum HDL-C levels can negatively impact lipid metabolism and overall health in animals [44]. Furthermore, we observed that the triglyceride and serum urea levels in the GFAE2 group were lower than those in the GFAE1 group, with no significant difference compared to the control group, suggesting that a high dose of GFAE may have caused an inhibitory effect. We hypothesize that higher concentrations of GFAE may alter the composition of the rumen microbiota, thereby affecting related parameters. Therefore, we will further investigate the regulatory mechanisms of GFAE on the rumen microbiota to better understand the complex effects of GFAE on energy and protein metabolism in beef cattle.

Rumen pH serves as a crucial metric for evaluating rumen health. In our study, rumen pH values ranged from 6.81 to 6.84, which falls within the healthy range for beef cattle (6.0–7.5) [45]. Volatile fatty acids (VFAs) are essential energy sources for ruminant growth, and VFAs produced through rumen fermentation provide sufficient energy and nutrients for ruminants [46]. Acetate is a key indicator for assessing rumen fermentation patterns and energy supply efficiency; higher acetate levels typically indicate efficient cellulose degradation, which is beneficial for energy supply and growth in beef cattle [47]. In this study, the levels of acetate, propionate, and total volatile fatty acids were notably greater in the experimental groups than in the control group. The higher propionate concentration indicates that beef cattle can more effectively utilize carbohydrates for energy metabolism. The aforementioned results align with those reported by Li et al. [48], who observed that dietary tributyrin supplementation increased the molar proportion of VFAs. The current research showed that supplementing with dietary GFAE notably raised the concentrations of acetate and propionate in the rumen, while not adversely affecting rumen health.

Bacteria, protozoa, and fungi are the three major microbial groups in the rumen digestive system [49]. These microbial communities promote the digestion and absorption of cellulose in feed by ruminants and synthesize microbial protein, which is a primary nutritional source for ruminants [50]. The addition of appropriate feed additives to the diet can promote the proliferation of beneficial rumen microbes, thereby enhancing animal growth rates and production efficiency [51]. In this experiment, the phyla Bacteroidota and Bacillota had the highest abundance, accounting for over 89% of the rumen microbial community. Bacteroidota is an important phylum in the rumen of ruminants, providing energy to the host and promoting muscle protein synthesis by degrading complex carbohydrates and synthesizing and metabolizing branched-chain amino acids [52]. Bacillota plays multiple key roles in the rumen of ruminants, including fiber degradation, short-chain fatty acid synthesis, maintenance of rumen health and fermentation balance, immune regulation, and resistance to environmental stress [53]. In the current study, the abundance of Fibrobacterota was notably higher in the GFAE1 group than in the control group. This conclusion aligns with Luan et al. [25], who reported that medium-chain glycerol fatty acid esters may increase Fibrobacterota abundance by modulating other microbes. This mechanism may involve glycerol fatty acid esters enhancing the concentration of metabolites such as L-threonine and 22-hydroxydocosanoic acid in rumen fluid, thereby creating a more favorable environment for Fibrobacterota growth. The reduction in the relative abundance of Fibrobacter in the high-dose group may be consistent with the findings of Hristov et al. [54], who discovered that the addition of excessive lauric acid to the rumen of dairy cows significantly decreased the number of rumen protozoa. This suggests that excessive SCFAs possess strong antimicrobial properties, thereby affecting the composition of the rumen microbial community. Fibrobacterota primarily degrades cellulose efficiently, promotes stable and efficient rumen fermentation, improves feed utilization, and interacts with other microbes to maintain rumen health in ruminants [55]. These functions are of critical importance for ruminant growth and production performance. In this study, incorporating GFAE at a dose of 0.1% into the diet significantly increased the relative abundance of Fibrobacterota.

Regarding the genus level, the predominant genera detected in this study encompassed *Prevotella*, *Bacteroides*, *Ruminococcus*, *Alistipes*, *Eubacterium*, *Clostridium*, *Sodaliphilus*, *Candidatus Cryptobacteroides*, *Aristaeella*, *Fibrobacter*, and some unclassified bacteria. Among them, Prevotella had the highest relative abundance, highlighting its important role in degrading lignocellulose in the rumen [56]. This high abundance of *Prevotella* is indicative of its significant contribution to the breakdown of complex plant materials, a process vital for efficient rumen fermentation and energy extraction in ruminants. Regarding genus classification, the GFAE1 group exhibited a notably higher proportional representation of *Bacteroides* and *Fibrobacter* compared to the con group. *Bacteroides* has strong fiber-degrading capabilities in the rumen [57]. These bacteria degrade complex plant cellulose and hemicellulose by secreting various cellulases and hemicellulases, promoting stable and efficient rumen fermentation. The abundance of *Bacteroides* is closely related to feed utilization efficiency in ruminants, and their metabolic activities in the rumen help enhance energy acquisition in ruminants [58]. Similarly, *Fibrobacter*, a key genus for cellulose degradation, is widely found in the gastrointestinal tract of ruminants and plays a crucial role in breaking down cellulose, thereby further improving feed utilization efficiency [59]. However, high doses of glycerol fatty acid esters may inhibit the enrichment of such beneficial bacteria. Supporting this conclusion is the finding by Burdick et al. [60]. The addition of excessive MCFA to the diet of lactating Holstein cows significantly reduced the abundance of *Butyrivibrio fibrisolvens* in the rumen and consequently led to a decrease in fiber degradation efficiency. The specific mechanism involves MCFA disrupting the integrity of cell membranes, thereby inhibiting the growth of fiber-degrading bacteria and reducing the secretion of cellulases and hemicellulases. Similarly, this phenomenon was also observed in the nutrient digestion trial. The digestibility of ADF and NDF was lower in the GFAE2 group than in the GFAE1 group. Therefore, we speculate that high doses of glycerol fatty acid esters in the diet can inhibit the abundance of fiber-degrading bacteria in the rumen, thereby affecting fiber digestibility. Both *Bacteroides* and *Fibrobacter* are potent fiber-degrading bacteria. The addition of glycerol fatty acid esters (GFAEs) at appropriate levels in the diet can promote the proliferation of these bacteria.

This study revealed a close association between rumen microbial composition and key fermentation parameters. It has been reported that the primary fermentation products of the genus *Aristaeella* include acetate, hydrogen, ethanol, and lactate [61]. The significant inverse relationship observed between *Aristaeella* and NH_3_-N indicates that this genus could be crucial in regulating ammonia metabolism within the rumen. Specifically, *Aristaeella* may participate in the reduction of NH_3_-N by converting ammonia nitrogen into other nitrogenous compounds that can be utilized for microbial growth or incorporated into microbial biomass [53]. Additionally, *Mogibacterium*, *Eubacterium*, and *Blautia* all exhibited significant negative correlations with ruminal NH_3_-N, suggesting that these bacterial genera may collectively participate in the utilization or transformation of NH_3_-N in the rumen. This conclusion aligns with the findings of Mlinar et al. [62], who demonstrated that specific strains of *Eubacterium* can utilize ammonia nitrogen as a nitrogen source for their growth, thereby lowering the levels of NH_3_-N in the rumen. *Mogibacterium* and *Blautia* are essential for the rumen microbiota, particularly in maintaining microbial stability and promoting the proliferation of beneficial bacteria. *Mogibacterium* is enriched in the gut microbiota of weaned lambs, enhancing microbial diversity and promoting the proliferation of beneficial bacteria, such as *Butyrivibrio* [63]. *Blautia* significantly influences rumen microbiota, particularly in producing and metabolizing short-chain fatty acids (SCFAs) [64]. Both beneficial bacteria and SCFAs play a crucial role in sustaining rumen pH stability, while the concentration of NH_3_-N is intricately linked to the rumen fermentation milieu. Consequently, we hypothesize that the enrichment of these genera may impact the variations in rumen NH_3_-N concentration. Regarding acetate metabolism, the significant negative correlations between *Clostridium* and *Aristaeella* with acetate suggest that these genera may be involved in acetate metabolism or may indirectly influence acetate production through synergistic interactions with other microbes. The genus *Aristaeella* may indirectly affect acetate concentration through its involvement in cellulose degradation and SCFA production [61]. Certain species of *Clostridium* in the rumen can degrade cellulose and hemicellulose into sugars, which are then fermented to produce short-chain fatty acids, with acetate being the predominant product [65]. The abundance of the genus *Saccharofermentans* is closely related to rumen pH and propionate concentration [66]. This study’s notable negative association between propionate and *Saccharofermentans* aligns with past research, reinforcing the critical function of *Saccharofermentans* in the fermentation processes of the rumen. Meanwhile, the significant positive correlations between rumen pH and *Eubacterium* and *Butyrivibrio* suggest that these genera may play important roles in maintaining rumen pH stability and optimizing the fermentation environment. *Eubacterium* and *Butyrivibrio* produce SCFAs such as butyrate and ammonia through the fermentation of dietary fiber and carbohydrates. These metabolic products can act as buffers in the rumen, thereby regulating rumen pH [67].

## 5. Conclusions

Dietary supplementation with glycerol fatty acid esters at 0.1% of the total diet improved the apparent digestibility of ADF and NDF, increased serum total antioxidant capacity, urea and triglyceride concentrations, elevated ruminal acetate, propionate, and total volatile fatty acids levels, increased the relative abundance of fiber-degrading bacteria, and enhanced average daily gain in beef cattle. Therefore, considering the outcomes of this research, incorporating 0.1% GFAE into the diet of beef cattle is advisable.

## Figures and Tables

**Table 1:** Ingredients and nutritional profile of the basal diet (on a dry matter basis).

Ingredients (%) DM	Percentage (%)
Corn Stover	2.66
Peanut Hay	2.72
Baijiu Distillers’ Grains	9.32
Corn Silage	10.2
Corn Germ Meal	30.8
Premix-Containing Concentrate Feed	44.3
Total	100
Nutrient levels	
NEg, Mcal/kg	1.22
DM	56.5
CP	11.7
NDF	56.8
ADF	25.4
EE	3.98

The nutritional composition of the premix containing concentrated feed on a DM basis was as follows: dry matter 91.97%, crude protein 27.18%, corrected starch 16.30%, neutral detergent fiber 19.98%, acid detergent fiber 6.37%, calcium 5.45%, and phosphorus 0.66%, 577.64 mg/kg of zinc; DM: dry matter, CP: crude protein, NDF: neutral detergent fiber, ADF: acid detergent fiber, EE: ether extract. NEg: net energy for gain. The Neg values were determined in accordance with NY/T 815 [18].

**Table 2:** Influence of GFAE on the growth performance of beef cattle.

Item	CON	GFAE1	GFAE2	SEM	*p*-Value
IBW, (kg)	514	513	496	9.59	0.70
FBW, (kg)	588	597	573	10.7	0.72
ADG, kg/d	1.23 ^b^	1.40 ^a^	1.29 ^ab^	0.03	0.04
DMI, kg/d	10.6	11.0	10.8	0.17	0.59
F/G	8.60	7.87	8.36	0.14	0.10

IBW: initial body weight; FBW: final body weight; ADG: average daily gain; DMI: dry matter intake; F/G: DMI/ADG. CON: 0%GFAE + TMR; GFAE1: 0.1%GFAE + TMR; GFAE2: 0.2%GFAE + TMR. Different letters indicate significant differences between the groups (*p* < 0.05).

**Table 3:** Impact of GFAE on nutrient digestibility in beef cattle.

Items	CON	GFAE1	GFAE2	SEM	*p*-Value
DM	81.6	83.3	82.7	0.34	0.14
CP	60.3	64.4	63.2	1.36	0.46
EE	83.6	85.4	85.5	0.36	0.18
NDF	56.1 ^b^	58.5 ^a^	56.9 ^ab^	0.38	0.02
ADF	34.9 ^b^	37.3 ^a^	35.7 ^b^	0.45	0.01

DM: dry matter; CP: crude protein; EE: ether extract; NDF: neutral detergent fiber; ADF: acid detergent fiber; CON: 0%GFAE + TMR; GFAE1: 0.1%GFAE + TMR; GFAE2: 0.2%GFAE + TMR. Different letters indicate significant differences between the groups (*p* < 0.05).

**Table 4:** The effect of GFAE on the serum biochemistry of beef cattle.

Items	CON	GFAE1	GFAE2	SEM	*p*-Value
MDA (nmol/mL)	2.73	2.73	2.89	0.08	0.71
T-AOC (U/mL)	10.5 ^b^	17.7 ^a^	18.0 ^a^	1.30	0.00
GPX (nmol/min/mL)	288	246	225	16.6	0.34
SOD (U/mL)	206	186	196	17.1	0.92
H_2_O_2_ (μmol/g)	1.63	2.14	1.79	0.21	0.67
TP (g/L)	79.6	87.7	70.0	4.39	0.29
ALB (g/L)	35.3	42.5	34.1	1.88	0.13
UREA (mmol/L)	2.00 ^b^	2.57 ^a^	2.20 ^b^	0.01	0.02
TG (mmol/L)	0.20 ^b^	0.26 ^a^	0.22 ^b^	0.01	0.02
TC (mmol/L)	5.46	5.41	5.63	0.07	0.41
HDL-C (mmol/L)	2.11 ^a^	2.19 ^a^	0.80 ^b^	0.27	0.02
LDL-C (mmol/L)	3.55	3.37	4.45	0.40	0.57
NEFA (mmol/L)	1.48	1.26	1.33	0.09	0.63

MDA: malondialdehyde; T-AOC: total antioxidant capacity; GPX: glutathione peroxidase; SOD: superoxide dismutase; H_2_O_2_: hydrogen peroxide; TP: total protein; ALB: albumin; UREA: urea; TG: triglycerides; TC: total cholesterol; HDL-C: high-density lipoprotein cholesterol; LDL-C: low-density lipoprotein cholesterol; NEFA: non-esterified fatty acid. CON: 0%GFAE + TMR; GFAE1: 0.1%GFAE + TMR; GFAE2: 0.2%GFAE + TMR. Different letters indicate significant differences between the groups (*p* < 0.05).

**Table 5:** Effects of GFAE on rumen fermentation parameters in beef cattle.

Items	CON	GFAE1	GFAE2	SEM	*p*-Value
pH	6.84	6.84	6.8	0.02	0.57
NH_3_-N, mg/100 mL	17.2	20	19.2	0.65	0.21
Acetate, mmol/L	55.5 ^b^	63.7 ^a^	63.1 ^a^	1.32	0.00
Propionate, mmol/L	13.6 ^b^	15.1 ^a^	14.8 ^a^	0.28	0.04
Butyrate, mmol/L	7.30	7.40	7.37	0.24	0.99
TVFA, mmol/L	76.3 ^b^	86.2 ^a^	85.3 ^a^	1.67	0.01
Acetate/Propionate ratio	4.10	4.22	4.26	0.06	0.51

TVFA: Total volatile fatty acid; CON: 0%GFAE + TMR; GFAE1: 0.1%GFAE + TMR; GFAE2: 0.2%GFAE + TMR. Different letters indicate significant differences between the groups (*p* < 0.05).

## Data Availability

The metagenomic sequencing raw data obtained from the rumen of Simmental crossbred beef cattle have been submitted to the NCBI Sequence Read Archive (SRA) database (Accession number: PRJNA1273287).

## Supplementary Materials

The following supporting information can be downloaded at: https://www.mdpi.com/article/10.3390/ani15152194/s1, Supplementary Table S1: Relative abundance of dominant phyla in cattle rumen fluid for different treatments (%); Supplementary Table S2: Relative abundance of dominant genera in cattle rumen fluid for different treatments (%). Supplementary Method S1: DNA Extraction and Sequencing and Sequencing Data Processing.
